# Ultrasensitive Molecular Detection at Subpicomolar Concentrations by the Diffraction Pattern Imaging with Plasmonic Metasurfaces and Convex Holographic Gratings

**DOI:** 10.1002/advs.202201682

**Published:** 2022-05-26

**Authors:** Mingxi Wu, Guohua Li, Xiangyi Ye, Bin Zhou, Jianhua Zhou, Jingxuan Cai

**Affiliations:** ^1^ School of Biomedical Engineering Sun Yat‐sen University Guangzhou 510275 China

**Keywords:** imaging‐based sensing scheme, metasurfaces, molecular detection, plasmonic

## Abstract

Compact and cost‐effective optical devices for highly sensitive detection of trace molecules are significant in many applications, including healthcare, pollutant monitoring and explosive detection. Nanophotonic metasurface‐based sensors have been intensively attracting attentions for molecular detection. However, conventional methods often involve spectroscopic characterizations that require bulky, expensive and sophisticated spectrometers. Here, a novel ultrasensitive sensor of plasmonic metasurfaces is designed and fabricated for the detection of trace molecules. The sensor features a convex holographic grating, of which the first‐order diffraction pattern of a disposable metasurface is recorded by a monochrome camera.The diffraction pattern changes with the molecules attached to the metasurface, realizing label‐free and spectrometer‐free molecular detection by imaging and analyzing of the diffraction pattern. By integrating the sensor with a microfluidic setup, the quantitative characterization of rabbit anti‐human Immunoglobulin G (IgG) and human IgG biomolecular interactions is demonstrated with an excellent limit of detection (LOD) of 0.6 pm. Moreover, both the metasurface and holographic grating are obtained through vacuum‐free solution‐processed fabrications, minimizing the manufacturing cost of the sensor. A prototype of the imaging‐based sensor, consisting of a white light‐emitting diode (LED) and a consumer‐level imaging sensor is achieved to demonstrate the potential for on‐site detection.

## Graphical abstract

A novel imaging‐based molecular detection platform is reported that integrates solution‐processed disposable metasurfaces with convex holographic gratings for trace amounts of molecule detection. The limit of detection (LOD) for the human Immunoglobulin G (IgG)IgG/anti‐IgG immune complex formation assay of the proposed sensor is 0.6 pm. This image‐based detection method is expected to be a promising technique for point‐of‐care and on‐site detection.

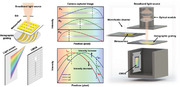

## 1 Introduction

Due to the unique capabilities of manipulating light scattering in the subwavelength regime, metasurfaces with metallic and dielectric nanophotonic structures have emerged as promising techniques in many nanophotonic applications^[^ ^1^, ^2^, ^3^, ^4^, ^5^, ^6^, ^7^ ^]^ The subwavelength light scattering of metasurfaces is highly localized in the vicinity of the nanostructure and is highly sensitive to the geometry of the structure and local environment, which makes them excellent for the refractometric sensing of thin‐layer substances attached to the nanostructures of metasurfaces.^[^ ^8^, ^9^, ^10^, ^11^, ^12^, ^13^ ^]^ Benefiting from the highly concentrated electric field on the subwavelength nanophotonic structures of metasurfaces, numerous dielectric and plasmonic metasurface‐based sensors have been intensively investigated for the detection of gaseous chemicals,^[^ ^14^ ^]^ biomolecules,^[^ ^12^, ^15^, ^16^ ^]^ environmental pollutants,^[^ ^17^, ^18^ ^]^ and corrosion.^[^ ^19^ ^]^ Generally, spectroscopic characterizations are utilized to record spectral changes of the metasurfaces, such as shifts in the resonant wavelength, caused by the existence of attached substances.^[^ ^20^, ^21^, ^22^, ^23^ ^]^ However, when the amount of attached substance is very small, high‐sensitivity and high‐resolution spectrometers are required to record the substance‐induced tiny spectral shifts. Such spectrometers are usually bulky, expensive and sophisticated, which hinders the application of metasurfaces in molecular sensing and many other daily detection scenarios.

Imaging‐based molecular detection based on metasurface devices through spectrometer‐free operations has recently been intensively investigated to overcome the aforementioned challenges. One of the most promising techniques is space‐multiplexed molecule detection based on metasurfaces with properly designed spatially varying nanostructures.^[^ ^24^, ^25^, ^26^, ^27^ ^]^ Because of the structure‐dependent scattering properties, the metasurface has structure‐dependent reflectance or transmittance under narrowband illuminations, which induces an intensity pattern. Therefore, attached analyte molecules on the metasurface alter the intensity pattern, which can be recorded by a camera and used to detect the existence of target molecules. Another imaging‐based molecular detection method is spectroscopic characterization of the reconstructed spectrum by imaging diffraction patterns of a plasmonic metasurface with an analyte substance attached.^[^ ^26^, ^28^, ^29^ ^]^ Although highly sensitive, the fabrication of such metasurfaces often involves an expensive and time‐consuming parallel electron beam lithography (EBL) process with a limited sensing area of a few hundred microns, as well as high wastage in material deposition.^[^ ^26^ ^]^ A microscopic setup is also required to analyze the tiny intensity pattern, which limits their further applications in practical molecular detection.

Here, we report a novel imaging‐based molecular detection platform that integrates solution‐processed disposable metasurfaces with convex holographic gratings for the ultrasensitive, label‐free, spectrometer‐free, and cost‐effective detection of trace amounts of molecules attached to a metasurface. The transmitted light of the disposable metasurface is efficiently diffracted by the convex holographic gratings and captured by a monochrome camera for intensity analysis. Attached or absorbed substances are detected by tracking the pixel shifts of the intensity distribution. An excellent limit of detection (LOD) of 4.3 × 10^−4^ RIU for refractometric sensing and an ultralow LOD of 0.6 pm for rabbit anti‐human immunoglobulin G (IgG) and human IgG immune complex formation analysis are reported. We also developed a prototype for compact and cost‐effective image‐based sensing based on a white light‐emitting diode (LED) and commercially available imaging sensors on mobile devices. This novel molecular detection platform is expected to be a promising technology for a broad area in point‐of‐care and on‐site applications.

## 2 Results and Discussion

**Figure 1:**
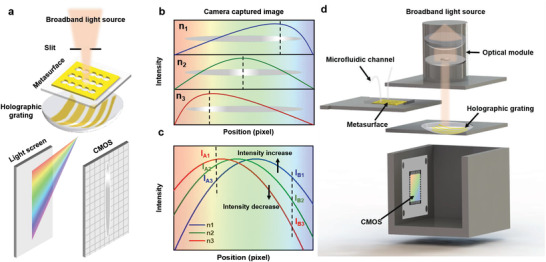
Principle of diffraction pattern imaging‐based molecular detection. a) Sketch of the imaging principle showing a sensing system illuminated with a broadband light source, an integration of plasmonic metasurface and convex holographic gratings, and the diffraction pattern on the light screen that is captured by an imaging sensor. b) The intensity of the diffraction pattern changes with the changes of refractive index of the covering medium. The dashed lines represent the highest intensity on the diffraction pattern. c) Diffraction intensity distribution curves extracted from (b). The shift of the maxima in intensity curves corresponds to the movement of the diffraction stripe. d) Schematic of a compact and cost‐effective imaging‐based sensing platform, consisting of an optical module including a white LED and a collimator, a microfluidic setup, a disposable metasurface, a holographic grating, and a commercial imaging sensor.

In the sensing scheme, the variation of spectral information induced by a local refractive index changes in the vicinity of the metallic nanostructures is mapped onto a diffraction intensity pattern by a convex holographic grating, enabling imaging‐based quantitative molecular detection. As illustrated in **Figure** **1**a, the detection platform consists of a broadband light source, a disposable metasurface, a convex holographic grating, and a monochrome camera. Specifically, the plasmonic metasurface is first illuminated by a beam of broadband light, the transmission of the metasurface is diffracted by the convex holographic grating and focused on an off‐plane screen, and the 1st order diffraction pattern is recorded by a monochrome camera. Analytes of different refractive indices adhered or adsorbed on the metasurface alter the transmission, as well as the diffraction intensity pattern, evidencing the existence of analyte molecules. We also developed an image processing algorithm to analyze the diffraction pattern to quantitatively detect the analyte molecules.

As illustrated in Figure S1, Supporting Information, the diffraction pattern relates to the transmittance spectrum of the metasurface. For a transmissive grating with normal incident, the grating equation is

(1) $$\sin\theta_{k}=k\frac{\lambda }{\Lambda }$$

where *k* is the diffraction order, *Λ* is the pitch of the grating, *θ_k_* is the diffraction angle, and *λ* is the wavelength of light.

According to Equation (1), a longer wavelength corresponds to a larger diffraction angle. Therefore, the intensity at different pixels in the camera‐captured diffraction pattern corresponds to the diffraction angle and wavelength of the transmittance spectrum of the metasurface. Since the light scattering properties of metasurface nanostructures are highly dependent on the local environment in the vicinity of the nanostructure, the diffraction pattern changes with the local refractive index (Figure 1b). For example, when the local refractive index increases from *n*_1_ to *n*_3_, the resonant peak shifts to longer wavelengths (Figure S2, Supporting Information), which leads to an intensity increase at pixel A and an intensity decrease at pixel B (Figure 1c). The intensity variations at each pixel constitute the changes in the intensity–position curve. By analyzing the changes in the intensity–position curve, the analyte change of refractive index induced by the can be quantitively detected. Figure 1d depicts a schematic diagram of the image‐based sensing platform integrated with a microfluidic setup, allowing accurate delivery of the target analyte. The incident light of a broadband light source in the optical module is collimated and illuminated on the disposable metasurface in a microchamber, diffracted by a holographic grating, and captured by a commercial complementary metal oxide semiconductor (CMOS). This integrated platform enables a cost‐effective, rapid, and accurate solution for on‐site refractometric sensing.

**Figure 2:**
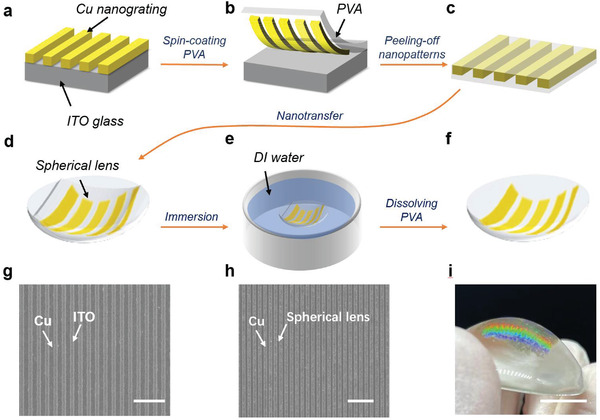
Nanotransfer fabrication of the convex holographic grating. a) An electrodeposited Cu nanograting on ITO glass. b) Spin coating of a thin layer of PVA aqueous solution on ITO glass. c) Peeling off the PVA with the embedded Cu nanograting. d) Attaching the PVA carrier onto a convex lens. e) Dissolving the intermediate PVA in DI water. f) The transferred nanograting is firmly attached on the convex lens. g) SEM image of an Au nanostructure on ITO. (h) and (i) SEM image and optical photograph of a Cu nanograting transferred to a lens. The scale bars in the SEM images and optical photograph represent 3 µm and 1 cm, respectively.

The holographic grating and disposable metasurface are fabricated through solution‐processed approaches modified from our previous publications.^[^ ^14^, ^25^, ^30^, ^31^ ^]^ Typically, the holographic grating is nanotransferred onto a spherical lens using polyvinyl alcohol (PVA) as the intermediate carrier. The nanotransfer process of the metallic grating is shown in **Figure** **2**. First, Cu nanograting is obtained on a conductive indium tin oxide (ITO) substrate via thermal nanoimprinting and electrodeposition. In the following nanotransfer process, PVA is spin‐coated onto the grating structure and peeled off from the ITO substrate with a Cu grating embedded in Figure 2b,c. Thereafter, a PVA film with an embedded nanograting is attached to the spherical lens and dissolved in deionized (DI) water, leaving the Cu grating attached to the surface (Figure 2d–f). Figures 2g,h show scanning electron microscopy (SEM) images of the Cu nanograting on the ITO substrate after the electrodeposition process and on the spherical lens after the nano‐transferring process, respectively. The photo of the diffraction of the holographic grating in Figure 2i indicates that the Cu nanograting was successfully transferred to the lens.

**Figure 3:**
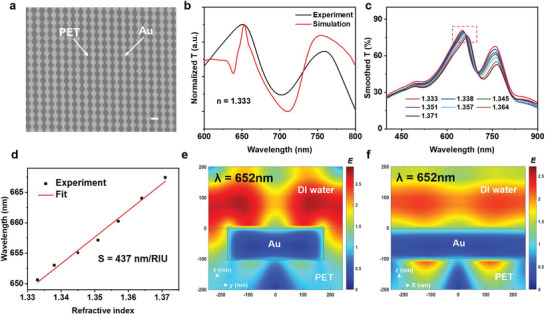
Morphological characterization and modeling of the optical properties of the plasmonic metasurface. a) SEM image of the metasurface with embedded Au nanostructures. The scale bar is 500 nm. b) Simulated and measured transmittance spectra of the metasurface covered by a medium with a refractive index of *n* = 1.333. c) Transmittance spectra of the metasurface with the increase in refractive index of the covered medium. d) Plot of the linear fitting of the intensity peak (labelled as dashed red box in (c)) and the refractive index. e,f) Electric field distribution of the metasurface at the wavelength of 652 nm from the longitudinal view.

The intensity distribution of the diffraction pattern is related to the analyte attached or adsorbed on the metasurface. The selection of resonant nanostructures is mainly considered from the following aspects in our molecular detection platform using diffraction imaging: first, the resonance should be located at the wavelength with high quantum efficiency of the CMOS to capture the diffraction pattern with low noise; second, the sensitivity of the metasurface should be high enough to ensure a visible changes in the intensity distribution of the diffraction pattern; and finally, the diffraction of the resonance should be projected to the light screen, which also restricts the design of resonance nanostructures. Therefore, to ensure a high sensitivity and signal‐to‐noise ratio, the plasmonic metasurface with resonant wavelength ≈600 to 700 nm was chosen here. The disposable metasurface with embedded Au nanoholes in a polyethylene terephthalate (PET) film is fabricated through nanoimprint lithography and electrodeposition processes. The whole fabrication process is illustrated in Figure S3, Supporting Information. **Figure** **3**a shows the SEM micrograph of the Au nanoholes embedded in PET film. Detailed characterizations of the fabrication process of metasurface are provided in Figure S4, Supporting Information. Figure 3b depicts the finite‐difference time‐domain (FDTD) simulated and experimentally measured transmittance spectra in the medium with a refractive index of 1.333. The simulated and measured transmittance spectra of the Au nanoholes are denoted as red and black curves, respectively. Notably, a feature corresponding to the Wood's anomaly occurs at a wavelength at 638 nm in the simulated spectrum but is not found in the measured spectrum, which might result from the imperfection of the solution‐processed fabrication. Most of the predicted resonant peaks and dips are found in the experimentally measured spectrum with slight shifts in the positions, which could be attributed to the dielectric constant difference between modeled and electroplated gold, in which impurity and roughness existed. Two resonance modes around *λ* = 652 nm and *λ* = 760 nm were found in the transmittance spectra of the metasurface at *n* = 1.333. Figure 3c shows the measured transmittance spectra of the plasmonic metasurface with different refractive indices ranging from 1.333 to 1.371, and a refractometric sensitivity of 437 nm RIU^−1^ of the resonance mode around *λ* = 652 nm is calculated (Figure 3d). The cross‐sectional view and top view of the electric field distributions around the nanoholes at *λ* = 652 nm with *n* = 1.333 obtained through FDTD simulations are shown in Figure 3e,f and Figure S5, Supporting Information, respectively, revealing that the enhanced electric field is highly confined to the surrounding outer volumes of Au nanoholes and tight near the top of the nanoholes. The sensing depth of the metasurface is ≈150 nm, which is overlapping with the absorbed or attached molecules, making the metasurface an excellent candidate for plasmonic molecular sensing applications.

**Figure 4:**
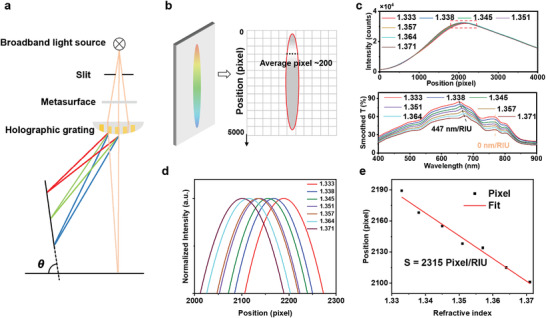
Demonstration of refractometric sensing and analysis of the limit of detection of the imaging‐based detection scheme. a) Schematic of the diffraction and focus of the plasmonic metasurface transmission by the holographic grating. b) Principle of imaging‐based molecular detection sensing platform. The intensity value is obtained by averaging a row of pixels in a row, as represented by the black dashed line. c) Diffraction intensity distribution (top) and transmittance spectrum (bottom) measured from the metasurface covered with medium of various refractive indices (*n* = 1.333 to 1.371). d) Normalized and zoomed‐in intensity curve around the maxima (labelled in (c) by the red dashed box). e) Plot of the intensity maxima shift in (d) versus the refractive index of covering medium. With linear fitting, the refractometric sensitivity of the imaging‐based scheme is 2315 pixels RIU^−1^.

The refractometric sensitivity of the imaging‐based sensing scheme is evaluated using the shift of the maxima in the diffraction intensity pattern induced by the changes in the transmission of the metasurface immersed in different refractive indices. The diffraction of the plasmonic metasurface transmission by the holographic grating is demonstrated in **Figure** **4**a. The holographic grating separates the incident light from the slit into component wavelengths and focuses the separated light onto the screen, thereby exhibiting an intensity pattern. The inclination of the screen *θ* relates to both the pitch of the holographic grating and the focal length of the spherical lens. For a pitch of 606 nm and a focal length of 14 mm, *θ* is close to 90°. To explain the effect of the angle more intuitively, we have added pictures of the diffraction pattern at *θ* = 0°, *θ* = 45°, and *θ* = 90°, as shown in Figure S6, Supporting Information. We can observe that the first‐order diffraction image is gently distorted at an angle of 45° and severely distorted at an angle of 0°. Hence, for the convenience of setup building, the screen is vertically mounted. As depicted in Figure 4b, the intensity as a function of the pixel of the camera‐captured image is determined by averaging hundreds of pixels in the row, which reduces nonsystematic errors and image noise created by the CMOS photons, resulting in an enhanced signal‐to‐noise ratio (S/N). A series of aqueous glycerol solutions with different refractive indices ranging from 1.333 to 1.371 is used as the covering medium of the metasurface to record both the transmittance spectra and diffracted images. The obtained results are summarized in Figure 4c. The emission spectrum of the broadband light source and the zero‐order transmittance spectra of the bare metasurface and the metasurface through the holographic grating are shown in Figure S7, Supporting Information, indicating that the variation of the zero‐order transmittance spectra through the holographic grating is corresponding to the resonance mode around *λ* = 652 nm. Figure 4d is the zoom‐in of the red dashed box in Figure 4c, which indicates that the maxima of diffraction intensity move toward the top of the image when the covering medium has a higher refractive index, representing the red‐shifts of resonant wavelength of the transmittance spectra in the bottom of Figure 4c. By fitting the pixel of the maxima of intensity–pixel curves, the sensitivity *S* is estimated to be 2315 pixel RIU^−1^ (Figure 4e). The LOD of refractometric sensing is calculated as *A/S*, where *S* is the sensitivity and *A* is the resolution of imaging, which is 1 pixel without further image processing algorithms. The refractometric LOD of this new imaging‐based sensing scheme is therefore determined to be 0.00043 RIU. As a comparison, the calculated spectral sensitivity of the disposable metasurface using the resonant peak at approximately *λ* = 652 nm obtained by a compact laboratory spectrometer (USB2000+, Ocean Insight) is 437.0 nm RIU^−1^, corresponding to a spectral LOD of 0.0034 to 0.00068 RIU at a typical spectral resolution ranging from 0.3 to 1.5 nm.

**Figure 5:**
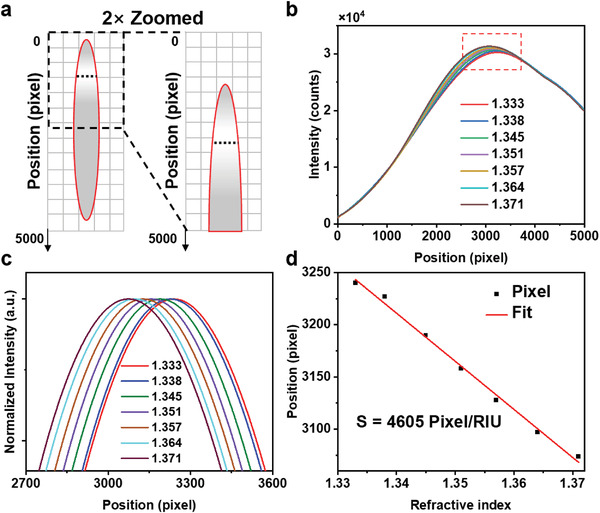
Improvement of refractometric sensing of environmental refractive index. a) Schematic of the zoomed diffraction pattern, which is half of the diffraction pattern. b) Diffraction intensity versus pixel of the zoomed diffraction pattern of metasurface covered with medium of various refractive indices ranging from 1.333 to 1.371. c) Normalized and zoomed‐in spectra of maxima in (b). d) Plot of the linear fitting of the intensity maxima shift versus the surrounding refractive index. The refractometric sensitivity is 4605 pixel RIU^−1^.

The sensitivity of our imaging‐based sensors can be further improved by increasing the movement of the diffraction intensity maxima in the captured image. According to the measurements, the diffraction pattern represents a spectral range of ≈400 to 900 nm, but the movement of the maxima only covers a small range. Therefore, optimization of the imaging by employing a 2× zoom lens to take the image of interest will further enhance the sensitivity by increasing the move of maxima (**Figure** **5**a). Moreover, the increase of averaging pixels in zoomed images also enhances the S/N of the platform. Figure 5b,c shows the diffraction intensity–pixel relationships obtained from 2× magnified images with covering medium of varying refractive indices (*n* = 1.333 to 1.371). The refractometric sensitivity retrieved from the zoomed images is 4605 pixel RIU^−1^ (Figure 5d), corresponding to an LOD of 0.00022 RIU, which is superior to that from spectroscopic characterization (0.0034 to 0.00068 RIU). Compared to spectrometer‐based sensors, the imaging‐based sensing scheme also features a high S/N, benefiting from the increased averaging pixels (200 to 300 pixels) compared to the linear charge‐coupled device (CCD) sensor array in spectrometers.

The reversibility and stability of our sensor are evaluated by switching the local refractive index. As shown in Figure S8, Supporting Information, we integrated a microfluidic setup with the metasurface and a commercial CMOS imaging sensor to record the diffraction pattern. The covering refractive index of the metasurface was switched between *n* = 1.400 and *n* = 1.333 every 60 s by successive injection of glycerol solutions with different concentrations into the microchamber. During three cycles, the average shift of the maxima is consistent with predictions, proving the excellent stability of our imaging‐based sensor. A supplementary video is also provided to demonstrate the dynamic changes of the diffraction pattern during experiments.

**Figure 6:**
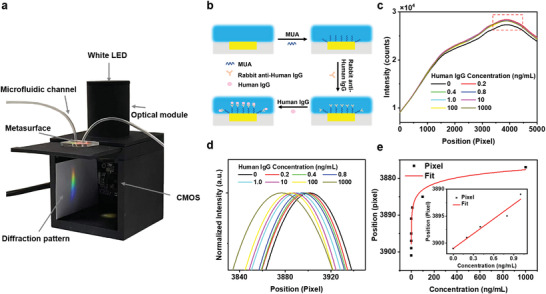
Demonstration of the detection of biomolecules. a) Photograph of a prototype of the compact and cost‐effective imaging‐based sensor. b) Schematic illustration of rabbit anti‐human IgG and human IgG interactions on the metasurface. c) Diffraction intensity distribution extracted from the diffraction images of the rabbit anti‐human IgG‐modified metasurface after specifically bonded with human IgG at different concentrations ranging from 0 to 1000 ng mL^−1^. d) Normalized and zoomed‐in curve of the maxima in (c). e) Measured position of the intensity maxima as a function of human IgG concentration. The red line is a Langmuir function fit to the human IgG concentrations. The inset shows the linear fit of the intensity maxima for IgG concentrations on a linear scale (0 to 1.0 ng mL^−1^).

**Figure** **6**a shows a prototype of a compact detection platform constructed based on our imaging‐based sensing scheme, incorporating an LED light source, a microfluidic setup integrated with disposable metasurface, a spherical holographic grating, and a commercial CMOS imaging sensor on mobile devices. This compact and cost‐effective detection platform is ideal for rapid and accurate on‐site sensing scenarios. Meanwhile, we also use this imaging‐based sensing scheme to quantitatively analyze IgG/anti‐IgG immune complex formation as a demonstration of biomolecular detection. The scheme of biomolecular detection is illustrated in Figure 6b. The microfluidic channel was first initialized with phosphate‐buffered saline (PBS) buffer solution. Then, the metasurface was modified with 11‐mercaptoundecanoic acid (MUA) and activated by 1‐ethyl‐3‐(3‐dimethylaminopropyl) carbodiimide (EDC) and N‐hydroxysuccinimide (NHS), respectively. The microchamber was flushed thoroughly with PBS solution before injection of anti‐IgG. Then, 200 µg mL^−1^ rabbit anti‐human IgG in PBS solution was injected into the microchamber to specifically bond the antibody on the metasurface. Once stabilized after 120 min, a resonant peak shift of 2 nm in the transmittance spectrum of the metasurface was observed, which revealed the saturated absorption of anti‐IgG molecules on the metasurface (Figure S9, Supporting Information). Thereafter, human IgG solutions at various concentrations ranging from 0.2 ng mL^−1^ to 1 µg mL^−1^ were injected into the microchamber for 30 min per incubation, allowing the sufficient specific binding of IgG and anti‐IgG. The unbound protein was removed by flushing with PBS solution, and the microchamber was thoroughly rinsed before injection of the IgG solution at a higher concentration. The specific binding of IgG and anti‐IgG on the metasurface leads to a shift in the diffraction intensity maxima, owing to the changes in the local refractive index around the metasurface. Figure 6c presents the diffraction pattern position versus diffraction intensity for binding under various IgG concentrations. As shown in Figure 6d, the binding of human IgG at concentrations of 0.2, 0.4, 0.8, 1, 10, 100, and 1000 ng mL^−1^ causes a shift in the intensity maxima of 2, 4, 6, 10, 13, 16, and 24 pixels, respectively.

Considering the reversibility of IgG/anti‐IgG binding, we applied the Langmuir isotherm model to quantitatively investigate our biosensor. Since the shift of the diffraction intensity pattern is linearly related to the shift of the spectral resonant peak, the Langmuir isotherm model can be expressed as:

(2) $$\Delta p=\Delta p_{\max}\frac{Kc}{1+Kc}$$

where *Δp*_max_ is the maximum (saturation) value of pixel shift *Δp*, K is the associated equilibrium constant, and *c* is the concentration of the human IgG solution. Figure 6e presents the fitting of the measured maxima position and the IgG solution concentration using the Langmuir model, from which the equilibrium constant K can be calculated to be 6.25 × 10^10^ m ^−1^, implying a good agreement with previous reports.^[^ ^32^ ^]^ The inset in Figure 6e presents a linear fit of the position of intensity maxima for IgG concentrations on a linear scale (0.2 to 1.0 ng mL^−1^). Hence, the sensitivity of the IgG/anti‐IgG assay on a linear scale was determined to be *S*_m_ = 9 pixel ng^−1^ mL. The LOD of the IgG/anti‐IgG assay was calculated as *A/S*_m_, where *S*_m_ is the sensitivity and *A* is the resolution of imaging. Considering that the resolution of imaging is 1 pixel, the LOD of the IgG/anti‐IgG assay is therefore determined to be 0.1 ng mL^−1^, corresponding to 0.6 pm.

To demonstrate the excellent performance of the diffraction imaging‐based detection platform, we performed a comprehensive comparison of the LOD and sensitivity of this novel platform with other recently reported methods that operated within the working spectral range of the silicon photodiode. As shown in **Table** **1**, the diffraction imaging‐based detection platform exhibits excellent sensitivity and LOD in both refractometric detection and IgG immunosensing while being more compact, indicating that this novel platform is expected to be a promising technique in biomolecular sensing applications.

**Table 1:** Comparison of the sensing performance of different detection systems

Author	LOD of RI	LOD of IgG	Sensitivity	Method
Funari et al.^[^ ^33^ ^]^	N/A	≈0.08 ng mL^−1^	183 nm/RIU	Spectrometer
Lu et al.^[^ ^34^ ^]^	N/A	1.1 nm	326 nm/RIU	Spectrometer
Cetin et al.^[^ ^28^ ^]^	≈4 × 10^−3^ RIU	N/A	621 nm/RIU	Spectrometer
Wang et al.^[^ ^35^ ^]^	N/A	60.7 pm	284 nm/RIU	Spectrometer
Hao et al.^[^ ^36^ ^]^	N/A	1.88 µg mL^−1^	273 nm/RIU	Spectrometer
Fernández et al.^[^ ^37^ ^]^	N/A	≈5.98 nm	587 nm/RIU	Spectrometer
Cao et al.^[^ ^38^ ^]^	N/A	≈0.4 nm	801 nm/RIU	Spectrometer
Xu et al.^[^ ^39^ ^]^	9.54 × 10^−8^ RIU	>12.6 pm	N/A	Spectrometer
Seiler et al.^[^ ^40^ ^]^	≈5 × 10^−4^ RIU	N/A	175 pixel/RIU	Image
Coskun et al.^[^ ^41^ ^]^	2 × 10^−3^ RIU	N/A	17.5392/RIU	Image
Cetin et al.^[^ ^42^ ^]^	N/A	0.4 ng mL^−1^	430 nm/RIU	Image
Min et al.^[^ ^25^ ^]^	9.6 × 10^−4^ RIU	N/A	1040 pixel/RIU	Image
This work	4.3 × 10^−4^ RIU	0.6 pm	4605 pixel/RIU	Image

## 3 Conclusion

In summary, we proposed an ultrasensitive, spectrometer‐free and cost‐effective sensing strategy using the diffraction pattern of metasurface transmission by a holographic grating through image acquisition and analysis. Our imaging‐based sensing platform has a refractometric LOD superior to that of the spectrometer‐based approach while being more compact and cost‐effective. We also demonstrated a real‐time assay of IgG/anti‐IgG immune complex formation with an excellent LOD of 0.6 pm. Incorporating a commercially available CMOS imaging sensor and white LED light source, this novel imaging‐based sensing scheme can also be further improved to achieve multiplexed detection, which is expected to be advantageous for cost‐effective point‐of‐care and on‐site detection applications.

## 4 Experimental Section

### Fabrication of Nanoimprint Template

A piece of 3 × 3 cm^2^ glass was ultrasonicated with isopropanol and acetone for 10 min before being dried with nitrogen flow and then cleaned with oxygen plasma for 5 min to make the glass surface hydrophilic. Then, 50 µL OrmoPrime (Micro Resist Technology) was spin‐coated on the glass substrate and baked on a hot plate at 150 °C for 5 min to increase the adhesion of the glass substrate. Next, 0.02 g OrmoStamp (Micro Resist Technology) was dropped on the silicon mold and left to stand for 5 min to allow the OrmoStamp to completely fill the nanostructures to form a uniform layer between the master silicon template and the glass substrate. After that, the stack was placed in a UV curing machine to cure the UV‐dependent resin. Finally, the imprint template was manually peeled off from the Si mold and baked at 150 °C for 20 min.

### Fabrication of the Disposable Metasurface

First, a layer of 72 nm‐thick thermal nanoimprint resist was spin‐coated on a cleaned conductive ITO glass substrate, and baked at 120 °C for 5 min and covered with the nanoimprint mold. The mold/substrate stack was heated to 100 °C for 5 min under a pressure of 0.3 tons to press the nanoimprinted template and ITO substrate by a hot embossing system (Specac). The pressure was maintained until the system was cooled to room temperature, and the ITO was peeled off from the mold, leaving nanostructured resist on the ITO substrate as an electrodeposition mask. Then, an Au electroplating solution (Caswell) was used for electrodepositing Au nanoholes on ITO through the exposed grooves in the mask using a home‐built electrodeposition system based on a source meter unit (Keithly 2450 SourceMeter) under a current density of 2.5 mA cm^−2^ for 3 min. Afterward, the sample was gently rinsed in acetone to remove the residual resist and then transferred to a 100 µm‐thick PET film by a thermal nanoimprint process using the Au nanoholes‐coated ITO as the nanoimprint mold. The detailed fabrication process can be found in Figure S3, Supporting Information.

### Fabrication of the Holographic Grating

First, an imprint resist patterned with 110 nm nanograting on an ITO substrate was obtained through thermal nanoimprint lithography. Then, Cu nanograting was electrodeposited on ITO through the exposed nanograting of the resist. Afterward, a layer of 10 µm‐thick PVA was spin‐coated on the sample and manually peeled off with the Cu grating embedded as the intermediate transfer film. The PVA film was then attached to a spherical lens with a focal length of 15 mm. The lens and PVA films were heated at 80 °C for 1 min to further enhance the adhesion. Finally, the lens was immersed in DI water for 12 h to fully dissolve PVA, leaving the Au nanograting successfully transferred and firmly attached to the spherical surface.

### Morphological Characterization

An SEM (ZEISS) was used to characterize the morphology of the samples.

### FDTD Simulations

Numerical simulation of the transmittance spectra and electric field distribution of the metasurface were performed using the commercial software FDTD Solutions (Lumerical Solutions Inc.). The geometry of the Au nanoholes used in simulations was measured from SEM images. The metasurface was illuminated with two orthogonally polarized plane‐wave light beams with wavelengths of 400–800 nm. The refractive index was 1.65 for PET, and the dielectric constant of Au was from the Palik model.

### Measurements of Transmittance Spectra

All transmittance spectra were collected using a standard laboratory spectrometer (USB2000+, Ocean Insight). The metasurface was illuminated under an unpolarized broadband light source.

### Image‐Based Sensing Setup and Signal Extraction

Broadband light was generated by a halogen lamp (HL2000, Ocean Insight) coupled to a multimode fiber (N.A. is 0.22, diameter of the core is 200 µm). The fiber was also employed as the slit. The diffraction images of metasurface transmissions on the vertical white screen were captured using a commercial monochrome CMOS image sensor equipped with an electric cooling system (QHY183M). A lens (Micro‐NIKKOR 55 mm f/2.8 AI‐s, Nikon) was applied in the optical path which included the exposure time of each image, which was composed of 5544 × 3694 effective pixels in 16 bits, was 400 ms (gain = 30), and the color space was MONO16. Intensity–pixel curves were extracted from the captured images, and the curves were smoothed to reduce the effect of fabrication defects.

### Refractometric Sensitivity Measurements

20 µL aqueous glycerol solutions with varying calculated concentrations was dropped on the metasurface, and a clean glass was used to cover the sample to help the liquid spread and maintain the same thickness during experiments. After each recording of the diffraction pattern, the metasurface was thoroughly rinsed with DI water and dried under compressed air flow.

### Biosensing Performance Measurements

The metasurface was integrated with a PDMS microchamber (inner volume: 8 mm × 8 mm × 0.15 mm) to assemble a microfluidic device for biosensing experiments. A liquid was injected through a tube by a syringe infusion pump (LongerPump) and flowed out from another tube. The microfluidic device was first initialized by injection of PBS buffer solution (Xiamen Haibiao Technology). Then, the metasurface was surface modified by the injection of 20 mm MUA solutions and incubation for 60 min. Afterward, a liquid composed of 200 mm NHS (Meryer) and 800 mm EDC (Beyotime) (1:1) was injected. After 30 min, rabbit anti‐human IgG (Beyotime) at a concentration of 200 µg mL^−1^ was injected. Two hours later, human IgG in PBS buffer solution with varying concentrations ranging from 0 ng ml^−1^ to 1 µg mL^−1^ was injected into the microfluidic chamber and allowed to stand for 30 min. After each image acquisition, the microchamber and tubes filled with low concentration human IgG were drained and then flushed with PBS buffer solutions for 5 min. Finally, the microfluidic device was drained again to be ready for the next higher concentration experiment.

## Conflict of Interest

The authors declare no conflict of interest.

## Supporting information

Supporting Information

Supplemental Video 1

## Data Availability

The data that support the findings of this study are available in the supplementary material of this article.

## References

[1] Z.‐L. Deng , G. Li , Mater. Today Phys. 2017, 3, 16.

[2] H.‐H. Hsiao , C. H. Chu , D. P. Tsai , Small Methods 2017, 1, 1600064.

[3] L. Jiang , S. Zeng , Z. Xu , Q. Ouyang , D. H. Zhang , P. H. J. Chong , P. Coquet , S. He , K. T. Yong , Small 2017, 13, 1700600. 10.1002/smll.201700600 28597602

[4] S. Tan , F. Yang , V. Boominathan , A. Veeraraghavan , G. V. Naik , ACS Photonics 2021, 8, 1421.

[5] A. Komar , R. A. Aoni , L. Xu , M. Rahmani , A. E. Miroshnichenko , D. N. Neshev , ACS Photonics 2021, 8, 864.

[6] Z. Li , X. Tian , C.‐W. Qiu , J. S. Ho , Nat. Electron. 2021, 4, 382.

[7] S. Nie , I. F. Akyildiz , Nat. Electron. 2021, 4, 177.

[8] M. Iwanaga , ACS Nano 2020, 14, 17458. 33231442 10.1021/acsnano.0c07722

[9] N. Bhalla , Y. Pan , Z. Yang , A. F. Payam , ACS Nano 2020, 14, 7783. 32551559 10.1021/acsnano.0c04421 PMC7319134

[10] M. Faraji‐Dana , E. Arbabi , H. Kwon , S. M. Kamali , A. Arbabi , J. G. Bartholomew , A. Faraon , ACS Photonics 2019, 6, 2161.

[11] M. L. Tseng , Y. Jahani , A. Leitis , H. Altug , ACS Photonics 2020, 8, 47.

[12] J. Zhu , Z. Wang , S. Lin , S. Jiang , X. Liu , S. Guo , Biosens. Bioelectron. 2020, 150, 111905. 31791874 10.1016/j.bios.2019.111905

[13] G. Palermo , K. V. Sreekanth , N. Maccaferri , G. E. Lio , G. Nicoletta , F. D. Angelis , M. Hinczewski , G. Strangi , Nanophotonics 2021, 10, 295.

[14] J. Cai , C. Zhang , A. Khan , C. Liang , W.‐D. Li , RSC Adv. 2018, 8, 5312. 35542388 10.1039/c7ra13516e PMC9078194

[15] Y. Wang , J. Zhou , J. Li , Small Methods 2017, 1, 1700197.

[16] Z. Long , Y. Liang , L. Feng , H. Zhang , M. Liu , T. Xu , Nanoscale 2020, 12, 10809. 32392273 10.1039/d0nr00288g

[17] I. Kim , W. S. Kim , K. Kim , M. A. Ansari , M. Q. Mehmood , T. Badloe , Y. Kim , J. Gwak , H. Lee , Y. K. Kim , J. Rho , Sci. Adv. 2021, 7, eabe9943. 33827821 10.1126/sciadv.abe9943 PMC8026120

[18] J. Siregar , N. L. W. Septiani , S. A. Abrori , K. Sebayang , Irzaman , M. Z. Fahmi , S. Humaidi , T. Sembiring , K. Sembiring , B. Yuliarto , J. Electrochem. Soc. 2021, 168, 027510.

[19] A. B. Tesler , T. Sannomiya , A. Vaskevich , E. Sabatani , I. Rubinstein , Adv. Opt. Mater. 2018, 6, 1800599.

[20] J. Cai , C. Zhang , C. Liang , S. Min , X. Cheng , W.‐D. Li , Adv. Opt. Mater. 2019, 7, 1900516.

[21] X. Hui , C. Yang , D. Li , X. He , H. Huang , H. Zhou , M. Chen , C. Lee , X. Mu , Adv. Sci. 2021, 8, 2100583. 10.1002/advs.202100583 PMC8373097 34155822

[22] S. Romano , G. Zito , S. Torino , G. Calafiore , E. Penzo , G. Coppola , S. Cabrini , I. Rendina , V. Mocella , Photonics Res. 2018, 6, 726.

[23] Y. Wang , M. A. Ali , E. K. C. Chow , L. Dong , M. Lu , Biosens. Bioelectron. 2018, 107, 224. 29475186 10.1016/j.bios.2018.02.038

[24] J. Bian , X. Xing , S. Zhou , Z. Man , Z. Lu , W. Zhang , Nanoscale 2019, 11, 12471. 31219124 10.1039/c9nr00455f

[25] S. Min , S. Li , Z. Zhu , Y. Liu , C. Liang , J. Cai , F. Han , Y. Li , W. Cai , X. Cheng , W. D. Li , Adv. Mater. 2021, 33, 2100270. 10.1002/adma.202100270 34085723

[26] F. Yesilkoy , E. R. Arvelo , Y. Jahani , M. Liu , A. Tittl , V. Cevher , Y. Kivshar , H. Altug , Nat. Photonics 2019, 13, 390.

[27] Y. Jahani , E. R. Arvelo , F. Yesilkoy , K. Koshelev , C. Cianciaruso , M. De Palma , Y. Kivshar , H. Altug , Nat. Commun. 2021, 12, 3246. 34059690 10.1038/s41467-021-23257-y PMC8167130

[28] A. E. Cetin , A. F. Coskun , B. C. Galarreta , M. Huang , D. Herman , A. Ozcan , H. Altug , Light: Sci. Appl. 2014, 3, e122.

[29] P. C. Biswas , S. Rani , M. A. Hossain , M. R. Islam , J. Canning , IEEE Sens. Lett. 2020, 4, 1.35582432

[30] J. Cai , C. Zhang , A. Khan , L. Wang , W. D. Li , ACS Appl. Mater. Interfaces 2018, 10, 28754. 30084253 10.1021/acsami.8b07411

[31] J. Cai , C. Zhang , W. D. Li , Adv. Opt. Mater. 2020, 9, 2001401.

[32] P. Gronski , R. Bauer , L. Bodenbender , E. J. Kanzy , K. H. Schmidt , H. Zilg , F. R. Seiler , Behring Inst. Mitt. 1988, 82, 127. 3408450

[33] R. Funari , K. Y. Chu , A. Q. Shen , Biosens. Bioelectron. 2020, 169, 112578. 32911317 10.1016/j.bios.2020.112578 PMC7467868

[34] M. Lu , H. Zhu , C. G. Bazuin , W. Peng , J.‐F. Masson , ACS Sens. 2019, 4, 613. 30698009 10.1021/acssensors.8b01372

[35] Y. Wang , L. Tang , Anal. Chim. Acta 2013, 796, 122. 24016592 10.1016/j.aca.2013.08.024 PMC3809993

[36] D. Hao , C. Hu , J. Grant , A. Glidle , D. R. S. Cumming , Biosens. Bioelectron. 2018, 100, 23. 28850824 10.1016/j.bios.2017.08.038

[37] F. Fernández , O. Garcia Lopez , E. Tellechea , A. C. Asensio , J. F. Moran , I. Cornago , IEEE Trans. Nanotechnol. 2014, 13, 308.

[38] J. Cao , E. K. Galbraith , T. Sun , K. T. V. Grattan , Sens. Actuators B 2012, 169, 360.

[39] C. Xu , G. Qiu , S. P. Ng , C.‐M. L. Wu , Ceram. Int. 2020, 46, 20993.

[40] S. T. Seiler , I. S. Rich , N. C. Lindquist , Nanotechnology 2016, 27, 184001. 27010077 10.1088/0957-4484/27/18/184001

[41] A. F. Coskun , A. E. Cetin , B. C. Galarreta , D. A. Alvarez , H. Altug , A. Ozcan , Sci. Rep. 2014, 4, 6789. 25346102 10.1038/srep06789 PMC4209447

[42] A. E. Cetin , S. N. Topkaya , ACS Photonics 2019, 6, 2626.

