# Supplementary material for: Ultrasensitive Molecular Detection at Subpicomolar Concentrations by the Diffraction Pattern Imaging with Plasmonic Metasurfaces and Convex Holographic Gratings
Source: Adv Sci (Weinh). 2022 May 26;9(22):2201682. doi: 10.1002/advs.202201682 (PMC9353501; doi:10.1002/advs.202201682)
Supplement: Supplementary file 1 — Supporting Information [file ADVS-9-2201682-s002.pdf]

Supporting Information for:

Ultrasensitive Molecular Detection at Subpicomolar Concentrations by Diffraction Pattern Imaging with Plasmonic Metasurfaces and Convex Holographic Gratings

Mingxi Wu ‡, *Guohua Li* ‡, *Xiangyi Ye, Bin Zhou, Jianhua Zhou**, *Jingxuan Cai**

‡ These authors contribute equally to this work

Mingxi Wu, Guohua Li, Xiangyi Ye, Bin Zhou, Dr. Jianhua Zhou, Dr. Jingxuan Cai

School of Biomedical Engineering, Sun Yat-sen University, Guangzhou, China, 510275

E-mail: caijx27@mail.sysu.edu.cn, zhoujh33@mail.sysu.edu.cn

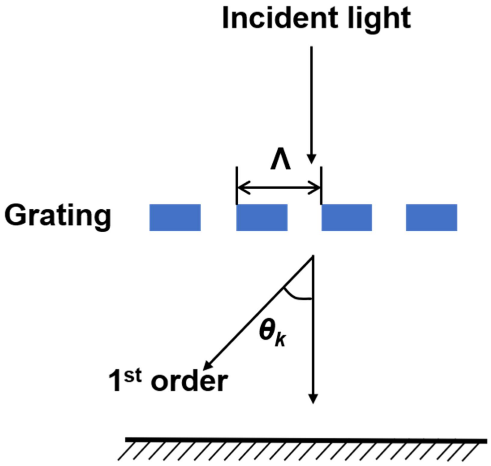

Figure S1. Schematic of the diffraction of nanograting. The angle of first-order diffraction can be described as $sin\theta_k = k\frac{\lambda}{\Lambda}$, where k is the diffraction order, Λ is the pitch of the grating, and λ is the wavelength of light.

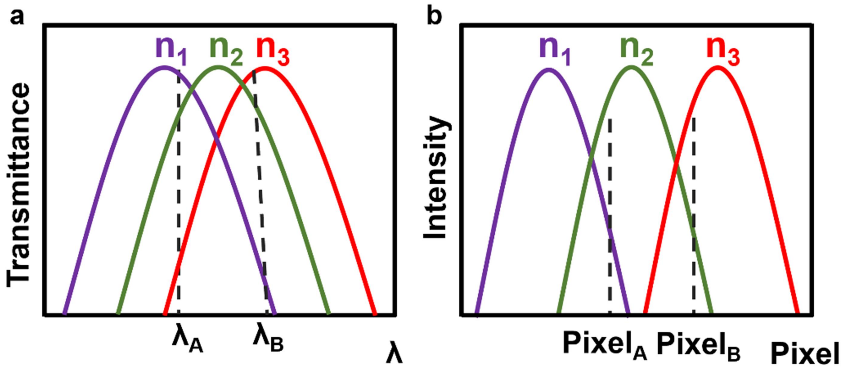

Figure S2. (a) When the local refractive index increases from n_1 to n_3, the resonant peak shifts to longer wavelengths, which leads to an intensity increase at pixel A and an intensity decrease at pixel B. (b) The diffraction intensity versus the position.

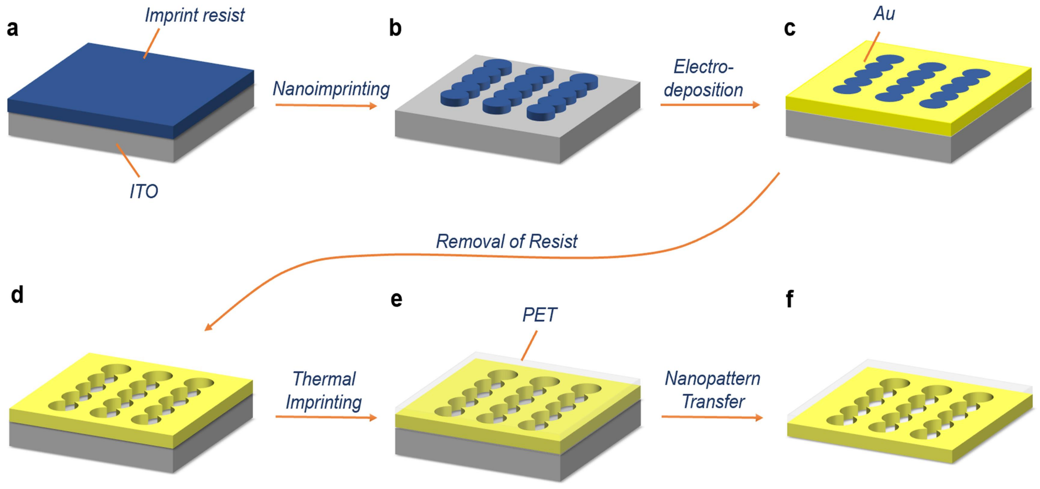

Figure S3. Schematic illustration of the fabrication process of the metasurface. (a) Spin coating of thermal imprint resist on ITO substrate. (b) Nanoimprinting on the thermal imprint resist. (c) Electrodeposition of gold inside the exposed grooves to form a complementary gold nanohole. (d) Removal of the resist. (e) Heating and pressing of the gold nanohole into a PET film. (f) Peeling off the PET film with the gold nanoholes transferred to and embedded in it.

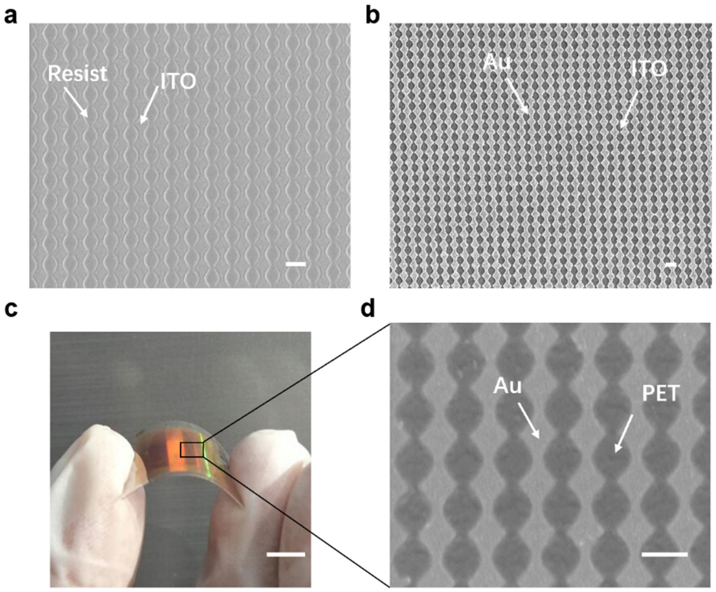

Figure S4. (a) SEM image of the resist on ITO by thermal nanoimprint. (b) SEM image of Au nanostructures on ITO. (c) Optical picture and (d) SEM image of the metasurface with embedded Au nanostructures. The scale bars of SEM and optical picture represent 500 nm and 1 cm.

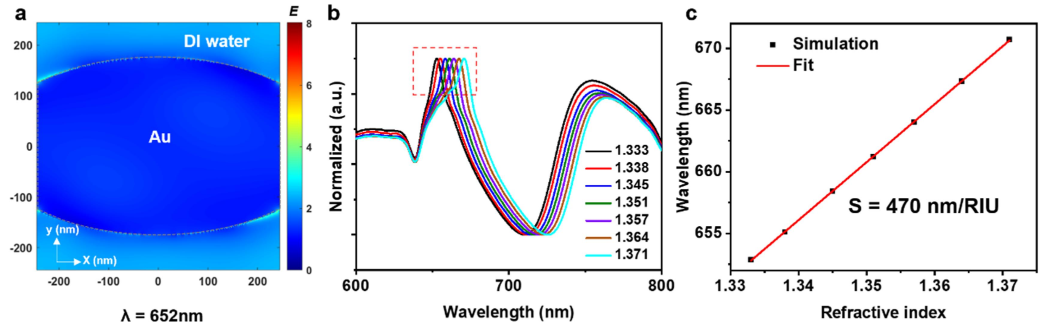

Figure S5. (a) Electric field distribution of the metasurface at the wavelength of 652 nm from the cross-section view. (b) The simulation spectrum of the plasmonic chip. (c) The sensitivity of the peak in (b).

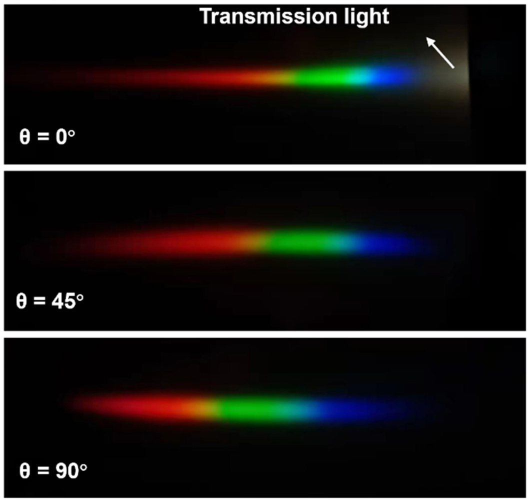

Figure S6. Diffraction images captured at different tilt angles of light screen at $\theta = 0°$, $\theta = 45°$, and $\theta = 90°$.

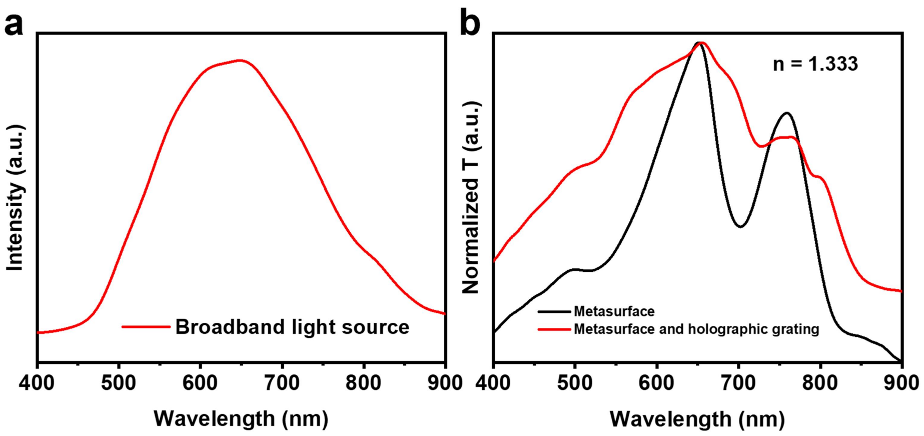

Figure S7. (a) The emission spectrum of the broadband light source. (b) Transmittance spectra of the bare metasurface (red line) and the metasurface through the holographic grating (black line).

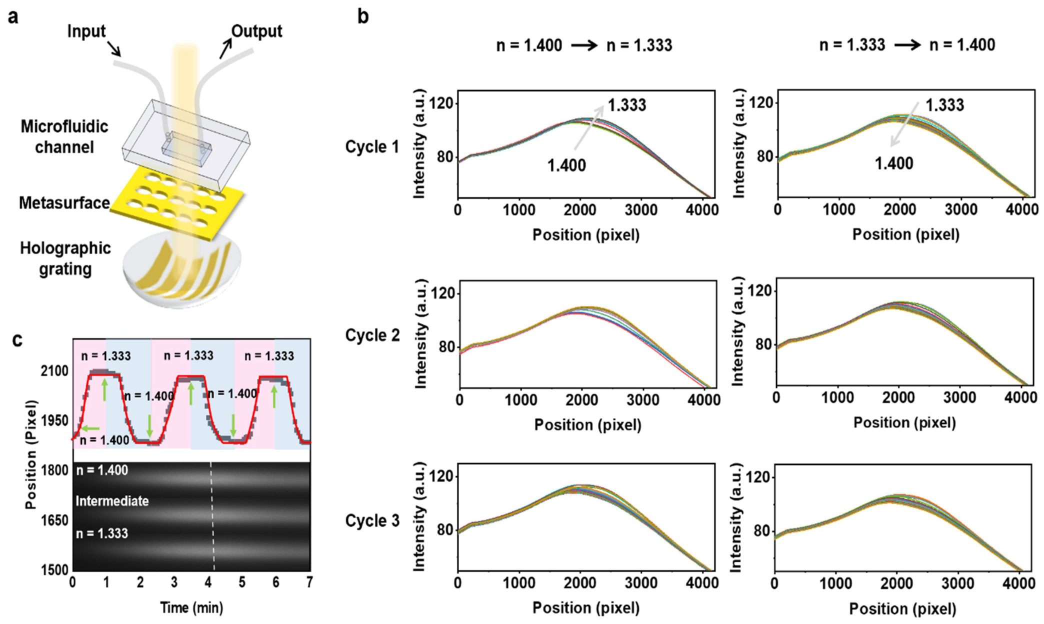

Figure S8. Real-time monitoring of glycerol solutions with different refractive indices ranging from 1.333 to 1.400. (a) Schematic of the detection platform. (b) Cyclic monitoring of environmental refractive index. The diffraction intensity distribution under various environmental refractive index was extracted from the recorded video. (c) The refractive index of covering medium is switched every 2 min by pumping the corresponding glycerol solution into the microfluidic chamber. (inset) Photographs of the diffraction pattern, the movement of the intensity maxima is depicted by the white dashed line.

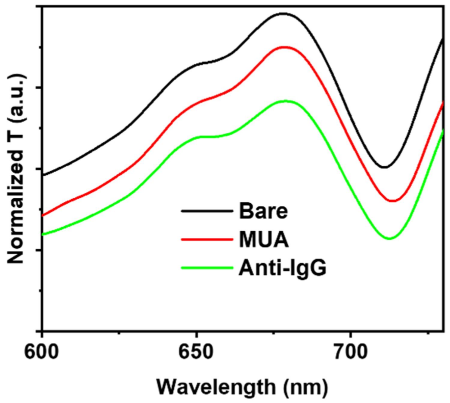

Figure S9. Transmittance spectra of the bare metasurface, MUA-modified metasurface, and rabbit anti-human IgG-modified metasurface under the environmental refractive index of 1.333.
